# Supplementary material for: Implementation of a smoking cessation intervention for people with severe mental illness in ambulatory mental healthcare (KISMET): A process evaluation
Source: PLoS One. 2025 Apr 29;20(4):e0322160. doi: 10.1371/journal.pone.0322160 (PMC12040152; doi:10.1371/journal.pone.0322160)
Supplement: S1 File — (DOCX) [file pone.0322160.s001.docx]

S1. Supporting Information

**Appendix I** Overview of KISMET group sessions

| **Weekly sessions** | **Content of sessions** |
| --- | --- |
| Session 1 | Introduction and personal motivation |
| Session 2 | Psycho-education and pharmacological treatment for smoking cessation (part I) |
| Session 3 | Psycho-education and pharmacological treatment for smoking cessation (part II) |
| Session 4 | Action plan and practical tips for a quit attempt |
| Session 5 | Relapse prevention and coping strategies |
| Session 6 | Pros and cons of smoking |
| Session 7 | Smoking and mental health problems |
| Session 8 | Recap of motivation and relapse prevention strategies |
| Session 9 | The function of smoking in emotion regulation |
| Session 10 | Smoking cessation and a healthy lifestyle |
| Session 11 | Smoking in relation with other substance use |
| Session 12 | Self-confidence and self-esteem |
| Session 13 | Reflection, CO monitoring and future goals |
|  |  |
| **Monthly sessions** |  |
| Session 14 – 20 | Recap of earlier sessions and new input based on patients’ experiences |

**Appendix IIa** Interview guide for interviews with patients

Motivation

1. How did you make the decision to (try to) stop smoking?
2. Have you made a quit attempt since you participated in the KISMET intervention?
   1. If yes: Did you experience slips or relapse?
      1. If yes: What happened when you relapsed?
      2. If no: Were there moments in which you experienced strong craving?
   2. If no: Can you describe why it was difficult for you to make a quit attempt?

The functional view on smoking

1. What does smoking mean to you?
   1. Which role does smoking have in your everyday life?
2. Do you ever experience feelings of loneliness?
   1. If so, does smoking play a role in having these feelings?
3. How do you think has your social environment influenced your smoking behaviour?

Experience with the KISMET intervention / Effectiveness

1. What effect do you think has the group on your attempt to quit smoking?
2. How did you experience it to be part of the group?
3. Did you feel supported by the other group members and the MHCPs?
4. If applicable: How did you experience participating in the group sessions after you had quit smoking?

Experience with the peer support group / Effectiveness

1. What did you think of the peer support group?
2. How did you experience the transition from weekly sessions to monthly sessions after three months?

Experiences with pharmacological treatment for smoking cessation / Effectiveness

1. Did you use pharmacological treatment? If so, could you describe how you made the decision to do so?
2. What effects did you experience (incl. side effects)?
3. Do you think you received sufficient information and support regarding pharmacological treatment?

Psychological and social factors and physical health

1. How did your social environment (friends/family/partner) respond when you started to participate in the KISMET intervention?
2. Did you notice a change in your mental health over the past year?
3. Did you notice a change in your physical health over the past year?
4. If applicable: How confident were you prior to the KISMET intervention that you could quit smoking?
5. If applicable: How confident are you now that you can quit smoking in the future?

General

1. How do you think can the KISMET intervention be improved?
2. Would you like to share anything else we have not talked about?

**Appendix IIb** Interview guide for interviews with mental healthcare professionals

Reach

1. How did you implement the recruitment of participants?
2. Do you feel that you have reached most patients whom you wanted to reach?
3. What challenges did you face during the recruitment phase and how did you deal with them?

Efficacy

1. How did you experience to lead the smoking cessation group?
2. What do you think of the psychological support for patients in the context of KISMET?
3. What do you think about peer support being included in the intervention?
4. How did you experience the prescription procedures regarding pharmacological support?
5. What did you think of the frequency and intensity of the group sessions? (Month 1-3: weekly; month 4-12: monthly)
6. What did you think about using the CO measurement?

Adoption

1. After the training, to which extent did feel well prepared to deliver the intervention?

Implementation

1. To which extent do you think the KISMET handbook offered you guidance? Did you deviate from the handbook and why?
2. Which challenges to implementing the intervention did you face and how did you deal with them?
3. What did you experience as facilitators to the implementation?
4. How did you experience your workload over the past 12 months? Did it influence the implementation?
5. How did you manage to integrate the KISMET intervention into your daily work?

Maintenance

1. Do you expect that the institution you currently work for will continue to offer smoking cessation support?

Motivation, attitudes and culture regarding smoking (cessation)

1. How did you and your colleagues make the decision to participate in the KISMET trial?
2. How was the motivation among patients in the group? Did it ever influence your own motivation as a professional?
3. How is the general culture regarding smoking within your institution and FACT team? Are your colleagues supportive of the KISMET intervention?
4. In your opinion, to what extent do you think a patient should take on ownership regarding their treatment? How do you evaluate the necessity to stimulate more at times?

**Appendix IV** Evaluation form on-site observations

Location:…….. Session number:……….. Date: dd/mm/yyyy
Observer:…….. Number of patients present: … out of …

1. **Mental healthcare professionals**
   1. How did mental healthcare professionals prepare before the start of the session?
   2. How was their contact with and attitude towards patients?
   3. How did mental healthcare professionals stimulate/engage patients during the session?
2. **Clients**
   1. Attitude: proactive or reserved? Were there differences between clients?
   2. Contact between patients and atmosphere: how did patients respond to each other?
   3. Did patients smoke in the break?
3. **Content and structure**
   1. Which topics were discussed during the session?
   2. To what extent did the content follow the handbook?

(completely / partly / not at all)

- 1. To what extent did the structure follow the handbook?
     (completely / partly / not at all)
  2. How did patients respond to the content and exercises of the session?
     1. Attention:
     2. Comprehension:
     3. Enthusiasm and involvement:
     4. Perceived usefulness for clients:

1. **Expert-by-experience**
   1. How was the interaction between patients and the expert-by-experience?
   2. Did the expert-by-experience explicitly hold positive or negative attitude towards smoking cessation?
2. **End of session**
   1. Was there enough time for patients to ask questions?
   2. Did patients immediately go out to smoke?
