# Supplementary material for: Implementation of a smoking cessation intervention for people with severe mental illness in ambulatory mental healthcare (KISMET): A process evaluation
Source: PLoS One. 2025 Apr 29;20(4):e0322160. doi: 10.1371/journal.pone.0322160 (PMC12040152; doi:10.1371/journal.pone.0322160)
Supplement: Attachment — Submitted filename: Response to Reviewers.docx [file pone.0322160.s003.docx]

*PLOS ONE*

Amsterdam, 26 February 2025

Dear dr. Ibrahim Jahun, dear Reviewers,

We would like to thank you for providing us the opportunity to revise and resubmit our manuscript entitled “Implementation of a smoking cessation intervention for people with severe mental illness in ambulatory mental healthcare (KISMET): a process evaluation”. We are pleased with the positive reviews and appreciate the recognition of the relevance and quality of our study.

Hereby, we send you our point-by-point response (see comments below) with page and line references to the manuscript file with tracked changes. We would also like to thank the editor and the two reviewers for their constructive and valuable feedback. We have revised our manuscript accordingly, and we feel that this has improved the quality of our paper considerably. We hope that our revised manuscript meets the requirements for being published in PLOS ONE and we look forward to your decision.

Best regards, on behalf of all co-authors,

Müge Küçükaksu

**Academic Editor**

**COMMENT 1:**

Methods – MHPs and patient selection and recruitment: “The selection of MHPs and patients is not clearly defined. Please provide inclusion and exclusion criteria for both MHPs and patients selection. This is in addition to the variations provided.”

**RESPONSE:**

Thank you for this comment. We added more details on how we selected MHPs and patients for the interviews.

**CHANGES TO THE MANUSCRIPT:**

Page 6, line 116-119: “To be eligible for an interview, patients and MHPs had to be part of FACT teams allocated to the intervention arm of the RCT, with patients still enrolled in the study and not having dropped out. MHPs had to be actively involved in providing the group or peer support sessions.”

**COMMENT 2:**

Methods – Data analysis: “Please clearly explain the approach used in coding the transcripts – inductive/deductive or both. Was coding framework developed? Etc.”

**RESPONSE:**

We have added details on how we approached data analysis using both inductive and deductive methods to generate an initial coding framework and (new) codes.

**CHANGES TO THE MANUSCRIPT**

Page 9, line 183-184: “Deductive and inductive thematic analysis was applied following six phases of analysis (Terry et al., 2017).”

Page 9, line 189-191: “Initially, a deductive method was applied to generate a coding framework based on the RE-AIM framework and the interview guide. During data analysis, an inductive method was additionally applied to generate new codes for new emerging relevant themes.”

**Journal Requirements**

**COMMENT 1:**

“Please ensure that your manuscript meets PLOS ONE's style requirements, including those for file naming.”

**RESPONSE:**

We made adjustments in line with PLOS ONE’s style requirements to (sub-)headings (bold, font size).

**COMMENT 2:**

“Please describe in your methods section how capacity to provide consent was determined for the participants in this study. Please also state whether your ethics committee or IRB approved this consent procedure. If you did not assess capacity to consent please briefly outline why this was not necessary in this case.”

**RESPONSE:**

Thank you for your comment. Capacity of patients to consent was assessed by mental healthcare providers while recruiting patients for the main trial, and ensured that capacity was maintained throughout the trial. Capacity for consent might be impaired in cases of severe psychotic symptoms. The Medical Ethical Committee of the Amsterdam University Centra approved the procedures of obtaining written informed consent, including the mental healthcare provider’s assessment of capacity to consent. Compliance with their guidelines concerning these procedures was a requirement for obtaining ethical approval for the study.

We added this information to the ‘Ethical considerations’ paragraph in the Methods section.

**CHANGES TO THE MANUSCRIPT:**

Page 10, line 205-208: “The information letter, the informed consent form and the procedure to obtain consent by signing an informed consent were approved by the Medical Ethical Committee of the Amsterdam UMC. Capacity (to consent) was assessed by qualified mental healthcare professionals.”

**COMMENT 3:**

“We note that the grant information you provided in the ‘Funding Information’ and ‘Financial Disclosure’ sections do not match. When you resubmit, please ensure that you provide the correct grant numbers for the awards you received for your study in the ‘Funding Information’ section.”

**RESPONSE:**

Thank you for bringing this to our attention. We have provided the grant number (258) for the primary funder in the ‘Funding Information’ section.

**COMMENT 4:**

“Thank you for stating the following financial disclosure: ‘This four-year research project is funded by the foundation Stichting tot Steun VCVGZ in the Netherlands.’

Please state what role the funders took in the study. If the funders had no role, please state: "The funders had no role in study design, data collection and analysis, decision to publish, or preparation of the manuscript."

If this statement is not correct you must amend it as needed.

Please include this amended Role of Funder statement in your cover letter; we will change the online submission form on your behalf.

**RESPONSE:**

We hereby confirm the following statement:

“The funders had no role in study design, data collection, analysis and interpretation, decision to publish, or preparation of the manuscript.”

Thank you for changing it in the online submission form on our behalf.

**COMMENT 5:**

“Before we proceed with your manuscript, please also provide non-author contact information (phone/email/hyperlink) for a data access committee, ethics committee, or other institutional body to which data requests may be sent. If no institutional body is available to respond to requests for your minimal data, please consider if there any institutional representatives who did not collaborate in the study, and are not listed as authors on the manuscript, who would be able to hold the data and respond to external requests for data access? If so, please provide their contact information (i.e., email address). Please also provide details on how you will ensure persistent or long-term data storage and availability.”

**RESPONSE:**

We have added a non-author institutional point of contact (phone/email) for requests to access to data to the ‘Availability of data’ statement at the end of the manuscript.

We have also added details on how we will ensure long-term data storage and availability.

**CHANGES TO THE MANUSCRIPT:**

Page 25, line 568-573: “Qualitative data of this study are not publicly available, due to their sensitive content and confidentiality. However, data can be made available by the corresponding author on reasonable request and by contacting dr. Barbara Groot-Sluijsmans ([secretariaat.agw.beta@vu.nl](mailto:secretariaat.agw.beta@vu.nl) ). To ensure long-term data storage and availability, we store the transcripts and analysis files on a password-protected drive to which only the research team and the non-author have access to.”

**COMMENT 6:**

“Your ethics statement should only appear in the Methods section of your manuscript. If your ethics statement is written in any section besides the Methods, please move it to the Methods section and delete it from any other section. Please ensure that your ethics statement is included in your manuscript, as the ethics statement entered into the online submission form will not be published alongside your manuscript.”

**RESPONSE:**

Thank you for this correction. We have moved the ethics statement (‘Ethical considerations’) to the Methods section.

**CHANGES TO THE MANUSCRIPT:**

Page 10, line 199-208: “Ethical considerations:

This study was carried out in line with the Declaration of Helsinki of 1975 (as revised in 2013), the Dutch Medical Research Involving Human Subjects Act (WMO), and the General Data Protection Regulation (GDPR). It has been approved by the Medical Ethical Committee of the University Medical Centres (UMC) in Amsterdam, Netherlands (NL76469.029.21, registered under 2021.0158). All interviewees received an information letter and, after seven days of time to consider, gave their written consent by signing an informed consent. The information letter, the informed consent form and the procedure to obtain consent by signing an informed consent were approved by the Medical Ethical Committee of the Amsterdam UMC. Capacity (to consent) was assessed by qualified mental healthcare professionals. Patients received a 10-euro voucher for their participation.”

**COMMENT 7:**

“Please include captions for your Supporting Information files at the end of your manuscript, and update any in-text citations to match accordingly.”

**RESPONSE:**

We have included captions for the Supporting Information file at the end of our manuscript (after references) and updated any in-text citations accordingly.

**CHANGES TO THE MANUSCRIPT:**

Page 28, line 680-681: “Supporting information: S1. Supporting information file”

**COMMENT 8:**

“Please review your reference list to ensure that it is complete and correct. If you have cited papers that have been retracted, please include the rationale for doing so in the manuscript text or remove these references and replace them with relevant current references. Any changes to the reference list should be mentioned in the rebuttal letter that accompanies your revised manuscript. If you need to cite a retracted article, indicate the article’s retracted status in the References list and also include a citation and full reference for the retraction notice.”

**RESPONSE:**

We have reviewed and adjusted our reference list, where needed.

**REVIEWER #1**

**COMMENT 1:**

The current project aimed to explore the perceptions of patients and mental health providers on the implementation of a smoking cessation intervention for individuals with severe mental illness. The mixed methods design provides critical insights for implementing smoking cessation interventions with difficult to treat populations. Overall, this very strong and sound study provides important information including the successes and difficulties of the KISMET program.

**RESPONSE:**

We thank the reviewer for this positive comment.

**COMMENT 2:**

“P3 L66-68 states that research on the combination of CBT, MI and NRT for smoking cessation is lacking; however several studies have begun looking at the effectiveness of these combined approaches (albeit in different populations, different timescales, and/or selective components of CBT, MI, pharmacotherapy) (see Brett et al., 2021; Okuyemi et al., 2013 Wittchen et al., 2011; Zhou et al., 2023.”

**RESPONSE:**

We thank the reviewer for this comment. We agree that there already are studies that shows the effectiveness of these approaches combined. We have adjusted this statement in our Introduction accordingly and added relevant references.

**CHANGES TO THE MANUSCRIPT:**

Page 4-5, line 65-77: “The effectiveness of cognitive behavioural therapy (CBT) and motivational interviewing (MI) techniques, combined with pharmacological support, such as nicotine replacement therapy (NRT) for smoking cessation in people with severe mental illness has been studied previously, studied previously, although not in combination with additional peer support (Rigotti et al., 2022; Loreto et al., 2017; Gilbody et al., 2019; Gilbody et al., 2015; Peckham et al., 2017; Spanakis et al., 2022; Zhou et al., 2023). However, the effectiveness of an intervention is not only based on its therapeutic quality, but also depends on its practical implementation. An optimal implementation within a mental healthcare organisation requires an interplay of multiple factors on different levels, such as prioritisation, allocation of financial resources to implement the intervention, sufficient available and qualified professionals, their affinity with smoking cessation, and effective teamwork. More evidence is needed on how the implementation of smoking cessation interventions in clinical practice can be promoted and optimised to ensure the best possible treatment effect.”

**COMMENT 3:**

“P20 L453-454 stated that shorter intervention periods may compromise the intervention effects. Brett et al (2021) found encouraging results for up to 6months after a single-session brief intervention. Although different, this suggests that shorter interventions may merit consideration and investigation, given the high drop out in the current project.”

**RESPONSE:**

We thank the reviewer for bringing this interesting point to our attention. We agree that a shorter intervention period might reduce dropout and could be a more effective option for some in line with bespoke care. However, we investigated the reasons for dropout and the duration of the study was not the most prominent reason for dropout. Interestingly, patients whom we interviewed agreed that the 3-months period with weekly sessions was experienced as too short. Moreover, the findings of our main trial support the notion that more sustained support can be beneficial, as some patients made a quit attempt towards the end of the 12-month intervention period.

**CHANGES TO THE MANUSCRIPT:**

Page 21, line 465-467: “However, the desired duration of the intervention can differ between individuals and future research could investigate the effectiveness of shorter interventions in this patient group.”

**COMMENT 4:**

“Finally, L480-481 discuses the impact of CO monitoring. The paper may benefit from research on other substance use on how self-monitoring can lead to reductions in substance use (e.g., Gass et al., 2021).”

**RESPONSE:**

We thank the reviewer for this comment and agree that the usefulness of self-monitoring in substance abuse in general adds to the argument in favour of CO monitoring in smoking cessation programs. We have added this to our discussion paragraph and referenced the mentioned systematic review.

**CHANGES TO THE MANUSCRIPT:**

Page 22-23, line 497-499: “These findings are in line with recent studies on the beneficial effect of self-monitoring on substance use (Gass et al., 2021), further advocating the use of CO monitoring to support smoking cessation.”

**References:** Gass, J. C., Funderburk, J. S., Shepardson, R., Kosiba, J. D., Rodriguez, L., & Maisto, S. A., (2021). The use and impact of self-monitoring on substance use outcomes: A descriptive systematic review. *Substance Abuse*, 42(4), 512-526.

**REVIEWER #2**

**COMMENT 1:**

This insightful study examines the implementation of the KISMET smoking cessation program in Dutch mental healthcare. Recruitment and retention posed significant challenges, with a high drop-out rate (58%) emphasizing the need for stronger motivational strategies. Patients found group and peer support valuable, though inconsistent attendance affected engagement. While pharmacological treatment was beneficial, better facilitation was needed, and staff shortages, along with limited collaboration, hindered implementation. The findings highlight the crucial role of teamwork, structured support, and effective supervision in strengthening smoking cessation programs for individuals with severe mental illness.

**RESPONSE:**

We thank the reviewer for this positive evaluation.
